# Cell-in-cell phenomena, cannibalism, and autophagy: is there a relationship?

**DOI:** 10.1038/s41419-017-0111-7

**Published:** 2018-01-24

**Authors:** Stefano Fais, Michael Overholtzer

**Affiliations:** 1 Department of Oncology and Molecular Medicine, National Institute of Health, Rome, Italy; 2 Cell Biology Program, Memorial Sloan Kettering Cancer Center, New York, NY USA

Phagocytosis is usually thought of as a process by which macrophages engulf unwanted material to clear it from the body, and possibly to present it to the immune system. However, what we consider as granted today was not so granted up to a century ago. In fact, Ilya Ilyich Metchnikoff (also written as Élie Metchnikoff), who received the Nobel Prize in Physiology or Medicine 1908, thought he had understood the function of phagocytes to include host defense and so expanded their aboriginal function from “eating to feed”, to “eating to defend”, thus becoming the real father of Immunity. Merging his interests in this protean cell with the newly discovered pathology of infectious diseases, Metchnikoff quickly developed a grand theory to account for the diverse functions of the phagocyte in development and in adult physiology. The so-called “phagocytosis theory” was presented to Rudolf Virchow in 1883, who apparently was favorably impressed, and Metchnikoff spent the rest of his career championing his grand synthetic theory. This notion is rooted in a century of nearly exclusive focus on the role of phagocytosis in immunity and the understanding of mechanisms involved in this process. Nevertheless, initial studies on phagocytosis, including those of Metchnikoff, stemmed from investigations of amoebas ingesting and feeding upon other microorganisms^1^. Only later was the existence of macrophages discovered in higher organisms. That phagocytosis in multicellular organisms can also be used for obtaining nutrients is apparent in the observation that tumor cells feed upon neighboring cells in a process called “tumor cell cannibalism”^2^, which is reminiscent of amoebal behavior.

There are different ways that cancer cells can cannibalize. One mechanism is referred to as cell cannibalism, where the active ingestion by cancer cells of other cancer cells, or of other cells in the microenvironment like immune cells, leads to engulfment and lysosomal digestion^2^. Another is called entosis, where the mechanism of engulfment may be different^3^, but the outcome is the same, as whole cells are ingested and degraded, and ultimately scavenged for nutrients to support proliferation. Other mechanisms called phagoptosis^4^, emperipolesis^5^, or others^6,7^ may share a similar outcome but involve different cell types.

It is becoming clear that cannibalistic mechanisms are regulated by nutrient signaling pathways. Entosis was recently shown to be induced to high levels by nutrient starvation, specifically by glucose withdrawal, in a manner involving activity of the AMPK kinase that is a known inducer of autophagy^8^. Entosis promoted proliferation and was required for population outgrowth under conditions of long-term starvation. Cell cannibalism is also known to be induced to high levels by nutrient starvation^9^, as metastatic melanoma cells were shown to ingest T cells at high rates when cultures were deprived of serum, and cannibalism in this context supported survival of the metastatic cells.

If cannibalistic activity can support the metabolism of starving cancer cells, is there a direct link to autophagy? Now a new study may have uncovered a direct link, as a protein called TM9SF4^10^, a nine transmembrane segment (TM9) protein previously shown to be required for phagocytosis in amoeba and *Drosophila* macrophages, and for the cannibalistic activity of malignant human cancer cells^11,12^, was identified as a regulator of autophagy. TM9SF4 localizes to lysosomes and was found to regulate autophagy initiation in response to nutrient starvation by inhibiting the nutrient-sensing kinase complex mTORC1. TM9SF4 silencing led to increased mTORC1 activity, reduced levels of autophagy, and decreased cell survival under starvation conditions, suggesting that this regulatory protein has key functions in nutrient homeostasis.

**Fig. 1:**
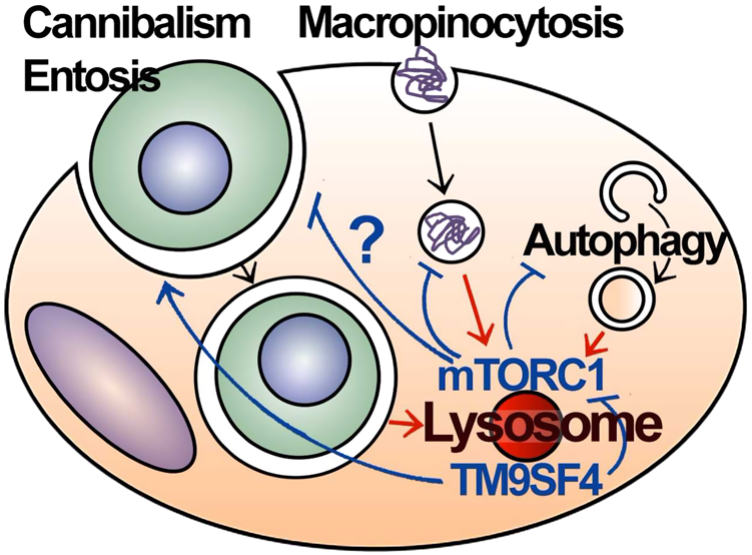
Regulation of mTORC1 and nutrient-scavenging pathways by TM9SF4 TM9SF4 localizes to lysosomes and negatively regulates mTORC1, leading to enhanced induction of autophagy upon nutrient starvation. TM9SF4 is known to promote cell cannibalism, and mTORC1 to suppress macropinocytic scavenging. Whether mTORC1 regulates cell cannibalism or entosis, and whether TM9SF4 regulates entosis, are unknown

What is the relationship between autophagy and cannibalism? It is possible that mTORC1 could act upstream to suppress both processes (Fig. 1). When nutrients are limited, a resulting decrease in mTORC1 activity leads to the induction of autophagy, and also has been shown to increase macropinocytic scavenging^13^, another endocytic mechanism that can support cancer cell metabolism, demonstrating that mTORC1 indeed suppresses an extracellular scavenging pathway under nutrient-replete conditions. TM9SF4 regulates both autophagy and cannibalism in a positive manner, and also contributes to suppressing mTORC1 activity in response to nutrient starvation. Further studies exploring the role of mTORC1 in cannibalism may shed light on whether this process is also directly regulated by this nutrient-sensing complex (Fig. 1). For example, are reduced rates of cannibalism in the absence of TM9SF4 reversed by inhibiting mTORC1?

Intriguingly, another direct link between autophagy and cannibalism is the cross-regulation of entosis by autophagy proteins, which function to enhance the digestion of engulfed cells. While the mTORC1-regulated autophagy-initiating ULK kinase complex is not required for entotic cell scavenging, the core machinery of autophagy, including Atg5 and Atg7, are involved, acting through a mechanism resembling what is referred to as LC3-Associated Phagocytosis^14,15^. Thus, while autophagy is constitutively suppressed by mTORC1 signaling under nutrient-replete conditions, bulk nutrient-scavenging through entosis occurs unabated. When entosis is induced by glucose starvation, in this context a known autophagy-activating kinase complex, AMP-regulated protein kinase (AMPK), is involved^8^. However AMPK activity is highest within the cannibalized cells, and AMPK is required specifically in this ingested cell population^8^, suggesting that those with the highest levels of autophagy may actually be sacrificed in the long-term to feed those with the lowest levels, a finding potentially relating to cell competition and selection for the fittest cells^16^.

Taken together, there is accumulating evidence that autophagy and cannibalistic mechanisms of nutrient-scavenging are indeed co-regulated. Scavenging mechanisms and autophagy are both induced under conditions of nutrient deprivation, and one key signaling node regulating both processes could be mTORC1 (Fig. 1). Other key questions remain. Do autophagy proteins contribute to the regulation of cell cannibalism? Why do autophagy proteins control entotic cell scavenging in an mTORC1 and ULK-independent manner? Is there competition for potentially limited autophagy machinery between nutrient scavenging and autophagy pathways? Are other mechanisms of whole-cell scavenging, such as phagocytosis, or emperipolesis, regulated by nutrient signaling?

One final aspect that may be involved in regulating the various engulfment processes exploited by cancer cells is the extracellular acidosis that is thought to represent a phenotype common to all cancers, which may also contribute to regulating autophagy. In fact, recent evidence suggests that autophagy in cancer is extremely dependent on microenvironmental pH^17^, and that induction of autophagy may represent an adaptation mechanism for cancer cells when exposed to an acidic environment^18^. Notably, an important difference between professional phagocytes, such as macrophages, and cancer cells is that, while they look very similar in the phagocytic process involving dead or amorphic material^19^, cancer cells exploit phagocytic process to feed, while macrophages also perform scavenging activity to clear debris and microorganisms^9^. This suggests that cancer cells represent in some way a reverse of Metchnikoff’s paradigm: from eating to defend to eating to feed. Milestone experiments have shown that whether you provide either cells or apoptotic bodies to starved cancer cells, they survive, while when you provide them with amorphic material, they die^9^.

All in all, more studies are needed to uncover the mechanistic parallels between phagocytosis, forms of cell cannibalism that occur in different cell types and contexts, and autophagy. While the role of autophagy in promoting the survival of starved normal cells is unquestionable, the role of the same phenomenon in cancer cells may be more complex. Similarly, cell engulfment by immune cells under normal conditions may be straightforward, but how forms of cannibalism in cancer cells are regulated under pathological conditions, and the consequences of these processes, may be more complex.
